# Multifunctional stimuli responsive polymer-gated iron and gold-embedded silica nano golf balls: Nanoshuttles for targeted on-demand theranostics

**DOI:** 10.1038/boneres.2017.51

**Published:** 2017-12-20

**Authors:** Liping Wang, Grace Jang, Deependra Kumar Ban, Vrinda Sant, Jay Seth, Sami Kazmi, Nirav Patel, Qingqing Yang, Joon Lee, Woraphong Janetanakit, Shanshan Wang, Brian P Head, Gennadi Glinsky, Ratneshwar Lal

**Affiliations:** 1 School of Biomedical Engineering, Shanghai Jiaotong Univerity, Shanghai, China; 2 Department of Mechanical and Aerospace Engineering, La Jolla, CA, USA; 3 Materials Science and Engineering Program, La Jolla, CA, USA; 4 Department of Nanoengineering, La Jolla, CA, USA; 5 Department of Chemical Engineering University of California, San Diego, La Jolla, CA, USA; 6 Department of Bioengineering, La Jolla, CA, USA; 7 Department of Anesthesiology, La Jolla, CA, USA; 8 Veterans Affairs San Diego Healthcare System, San Diego, CA, USA; 9 Institute of Engineering in Medicine, La Jolla, CA, USA

## Abstract

Multi-functional nanoshuttles for remotely targeted and on-demand delivery of therapeutic molecules and imaging to defined tissues and organs hold great potentials in personalized medicine, including precise early diagnosis, efficient prevention and therapy without toxicity. Yet, in spite of 25 years of research, there are still no such shuttles available. To this end, we have designed magnetic and gold nanoparticles (NP)-embedded silica nanoshuttles (MGNSs) with nanopores on their surface. Fluorescently labeled Doxorubicin (DOX), a cancer drug, was loaded in the MGNSs as a payload. DOX loaded MGNSs were encapsulated in heat and pH sensitive polymer P(NIPAM-co-MAA) to enable controlled release of the payload. Magnetically-guided transport of MGNSs was examined in: (a) a glass capillary tube to simulate their delivery via blood vessels; and (b) porous hydrogels to simulate their transport in composite human tissues, including bone, cartilage, tendon, muscles and blood–brain barrier (BBB). The viscoelastic properties of hydrogels were examined by atomic force microscopy (AFM). Cellular uptake of DOX-loaded MGNSs and the subsequent pH and temperature-mediated release were demonstrated in differentiated human neurons derived from induced pluripotent stem cells (iPSCs) as well as epithelial HeLa cells. The presence of embedded iron and gold NPs in silica shells and polymer-coating are supported by SEM and TEM. Fluorescence spectroscopy and microscopy documented DOX loading in the MGNSs. Time-dependent transport of MGNSs guided by an external magnetic field was observed in both glass capillary tubes and in the porous hydrogel. AFM results affirmed that the stiffness of the hydrogels model the rigidity range from soft tissues to bone. pH and temperature-dependent drug release analysis showed stimuli responsive and gradual drug release. Cells’ viability MTT assays showed that MGNSs are non-toxic. The cell death from on-demand DOX release was observed in both neurons and epithelial cells even though the drug release efficiency was higher in neurons. Therefore, development of smart nanoshuttles have significant translational potential for controlled delivery of theranostics’ payloads and precisely guided transport in specified tissues and organs (for example, bone, cartilage, tendon, bone marrow, heart, lung, liver, kidney, and brain) for highly efficient personalized medicine applications.

## Introduction

The controlled delivery of active molecules in specific cells and tissue is highly challenging. It becomes more difficult to deliver drug and active biomolecules in highly vascularized and hierarchical structure such as bone and cartilages. However, the development of multifunctional integrated nanomaterials with magnetic, optical and electronic properties have opened new avenues in nanomedicine.^1–5^ In bone tissue, nanomaterials can be used for drug and biomolecule delivery, tissue repair, and differentiation of stem cells to osteocytes.^6^ Multifunctional nanostructures may deliver drugs and active components for bone tissue repair. The incorporation of nanoparticles in scaffolds for bone tissue improves their efficiency and delivers the drug and gene in a regulated manner for treatment of bone related diseases.^7,8^ Therefore, nanomaterials can be used to design smart nanoshuttles for targeted delivery of biomolecules for diagnosis and therapy (theranostics)^9–13^ with improved clinical efficacy and lower toxicity.

Nanoscale drug delivery systems under development and evaluation use various basic materials, including magnetic NPs,^14,15^ nanogold structure,^16,17^ nanosilica structures,^18^ nanocarbons,^19^ stimuli-responsive polymer moieties,^20^ metal NPs ^21^ and semiconductor NPs.^22^ Gold and iron NPs have attracted much attention in theranostic applications because of their biocompatibility and multifunctional characteristics. Gold NPs can be tuned to exhibit distinctive optical properties in near-infrared (NIR) region that allow photothermal therapy as well as localized imaging-based diagnosis.^23,24^ Super paramagnetic properties of iron oxide NPs are suitable for magnetically targeted delivery of therapeutic molecules and holds significant potential for clinical applications.^25,26^

We reasoned that a hollow nanoshuttle made of hybrid materials, such as silica, gold and iron oxide NPs with multimodality functions would have broad applications in personalized nanomedicine ranging from imaging to therapy. To this end, we integrated the gold and iron oxide NPs in the hollow silica golf balls (termed MGNS) as a next generation multifunctional delivery system. In order to control on-demand delivery by external physio-chemical stimuli, we enclosed MGNS in heat and pH sensitive polymer P(NIPAM-co-MAA) as a gatekeeper. To examine the effectiveness of P(NIPAM-co-MAA) coated MGNS for target on-demand payload delivery, we loaded Doxorubicin (DOX), a known cancer drug, in the MGNS using precipitation method. Further, we investigated the drug release in epithelial and neuronal cells—the HeLa cells and differentiated human neuronal progenitor cells (NPCs) derived from induced pluripotent stem cells (iPSCs).^27^ The results of fluorescence imaging experiments showed a controlled DOX release as a function of pH and temperature. The transportation of MGNS was demonstrated in simulated capillary flow and in porous tissue models under external magnetic field using 5% polyacrylamide gel (PAG). Atomic force microscopy successfully showed the cellular uptake of MGNS and viscoelasticity of PAG in tested environment. The results of the studies delineate potential of MGNS for theranostics in bone, muscles, brain and other human tissues and organs.

## Materials and methods

### Materials

The aqueous suspension of carboxylated polystyrene microspheres of average size 0.2 μm and 0.05 μm and poly(diallyldimethylammonium chloride) (PDDA) were purchased from PolySciences, Inc, ammonium hydroxide (NH_4_OH), potassium carbonate (K_2_CO_3_), isopropyl alcohol (IPA), and ammonium carbonate were procured from Fisher Scientific, NJ, USA. Tetraethyl orthosilicate (reagent grade, 98% (TEOS)), chloroauric acid (HAuCl_4_), tetrakis(hydroxymethyl)phosphonium chloride (80% in water (THPC)), sodium hydroxide (NaOH), 3-[4,5-dimethylthiazol-2-yl]-2,5-diphenyltetrazolium bromide (MTT), doxorubicin hydrochloride (DOX), dimethyl sulfoxide (DMSO), P(NIPAM-co-MAA) formaldehyde solution (16%) were purchased from Sigma-Aldrich (St. Louis, MO, USA); Dimethylformamide (DMF) was purchased from Macron Chemicals, PA, USA; iron oxide nanoparticles with carboxylic acid group were purchased from Ocean NanoTech, SD, USA. Anhydrous ethyl alcohol (EtOH) was purchased from JT Baker, NJ, USA. Borosilicate Capillary tube (0.86 mm OD) was obtained from Warner Instruments, CT, USA and polydimethylsiloxane (PDMS) Sylgard 184 silicone elastomer kit from Dow Corning, Auburn, USA.

### Carboxylate-modified polystyrene core and satellite template formation

The core template was synthesized by mixing of 3.5 mL of 0.35% (w/v) 0.2 μm diameters carboxylate polystyrene beads with 2.5 mL of 1 wt% PDDA and stirred for 20 min. The excess material was removed by washing template by centrifugation at 10 000 *g* for 40 min with DI water and at 8 000 *g* for 10 min with EtOH. PDDA-modified polystyrene beads template was redispersed in 5 mL of EtOH.

Core-satellite-based polystyrene template was prepared by mixing 1 mL of 0.25% (w/v) aqueous suspended carboxylate-modified polystyrene beads of the average size of 0.05 μm on PDDA functionalized core particle solution. The mixed solution (core-satellite template) was tubmled overnight to perform the electrostatic attachment of negatively charged 0.05 μm beads on the PDDA-functionalized polystyrene core solution. After overnight incubation, the solution was treated with the 254 nm UV irradiation for 30 min and was vortexed well before attaching gold and iron oxide nanoparticle to the template.^28^

### Synthesis of gold nanoparticles

The gold nanoparticle (AuNP) with net negative charge was prepared by adding 12 μL of 80% THPC solution in 1 mL of water and stirred for 5 min. After diluted THPC solution and 50 μL of 10 mol·L^−1^ NaOH were added to 54 mL of an aqueous solution, the mixed solution was stirred for 5 min, and 2 mL of 1 wt% HAuCl_4_ was added. The final solution was stirred for another 30 min and stored at 4 °C at least for 24 h.

### AuNP and iron oxide nanoparticle attachment

The carboxylic acid functionalized iron oxide nanoparticles of average size of 0.01 μm and 0.003 μm AuNP were added to make the nano golf ball with tracking and magnetic targeting property. The electrostatic attachment of negatively charged iron oxide nanoparticle (IONP) and AuNP on the positively charged PDDA-modified polystyrene spheres was performed. Initially, 50 μL of IONP (0.01 μm, Ocean Nanotech) was added in 5 mL of PDDA modified core-satellite template solution and tumbled in room temperature for 3 h. After incubation, 2.5 mL of AuNP was added, and the template was stirred at 60 °C for overnight. The sample was rinsed twice with water at 3 200 *g* for 30 min and redispersed in 5 mL of 80% Ethanol.

### Growing silica on surface

TEOS was used to grow the silica on the IONP and AuNP attached core-satellite template. For the synthesis, 5 mL of IONP and AuNP attached template particles were added to 17.5 mL of IPA. Subsequently, 4.5 mL of water and 0.15 mL of ammonium hydroxide were mixed and sonicated for 5 min. Finally, 4.5 mL of 0.2% (v/v) TEOS in EtOH was added using a syringe pump with a rate of 0.2 mL·min^−1^ and stirred for 20 h at room temperature to allow the silica coated templates to settle down.

### Magnetic and gold particles embedded nanoshuttles formation

The MGNSs were prepared by etching the polystyrene templates. The MGNS suspension was rinsed twice with EtOH at 3 200 *g* for 20 min. Polystyrene spheres were removed by adding 5.7 mL of DMF to the MGNS suspension and incubated for 40 h at 60 °C with continuous stirring. After the polystyrene spheres had been etched, MGNS was rinsed with EtOH twice.

### Loading of DOX in the MGNS

1 mg of DOX was added to 1 mL of PBS (pH=7.4).After DOX was dissolved in the PBS, 100 μL of the MGNS was added into DOX solution. Then the solution was stirred for 30 min, and 1 mL of ammonium carbonate (0.1 M) was added into DOX solution. The mixed solution was stirred at room temperature for overnight. The solution was covered with aluminum foil to prevent photobleaching. The solution was centrifuged at 10 000 *g* for 30 min; the collected MGNS with DOX was rinsed three times at 10 000 *g* for 30 min. Then the collected MGNS with DOX was dried at room temperature. The dried MGNS with DOX was redispersed in 1 mL PBS, and the 1 mg of P(NIPAM-co-MAA) was added into the solution. After the solution was stirred overnight at room temperature, the golf ball covered with polymer was collected at 10 000 *g* for 30 min and rinsed three times at 10 000 *g* for 30 min. The collected sample was dried under vacuum at room temperature.

### Drug loading and release analysis

The amount of DOX-loaded in MGNS was determined by the fluorescence spectrophotometer analysis of the sample by exciting at 505 nm and collecting emission at 559 nm with fix emission and excitation slit width of 5 nm. The drug loaded MGNS sample was named as MGNS/DOX. The loading efficiency (LE %) of DOX was calculated by using following equation:

$$\text{LE}\%=\frac{W_{i}-W_{r}}{(W_{i}-W_{r})+W_{\mathrm{MGNS}}+W_{\mathrm{Poly}}}x100$$

Here, *W*_i_ is initial amount of Dox; *W*_r_ is a residual amount of DOX; *W*_MGNS_ is amount of MGNS; *W*_Poly_ is amount of P(NIPAM-co-MAA). The experiment was repeated three times.

The thermal and pH stimuli dependent control release was investigated by analyzing the release profiles of DOX from MGNS/DOX at 25 °C, 35 °C, 37 °C, and 42 °C in certain media solutions (pH 5.5, 6.8 or 7.4, phosphate buffer solution). Briefly, after 2 mg of DOX-loaded polymer covered MGNS was dispersed in 2 mL of media solution, at specified intervals, the solution was centrifugated for 30 min at 10 000 *g*, and the supernatant was taken out to determine the DOX release amount from MGNS/DOX by fluorescence spectrum with ex at 505 nm and em at 559 nm.

### Magnetic field-guided MGNS transport

External magnetic field-guided transport of MGNS in 5% polyacrylamide gel and capillary tubes was performed to simulate the transport of MGNS/DOX in porous bone like material and blood capillaries. The gel porosity and Young’s modulus were calculated using AFM analysis. Capillary tube (ID 0.86 mm) was washed in piranha solution prior to being embedded in a polydimethylsiloxane (PDMS) gel before MGNS transport analysis. PDMS made from Sylgard 184 silicone elastomer kit (Dow Corning) was cured at 80 °C for two hours. We have added 10 μL of 4 mg·mL^−1^ MGNS in PBS to a capillary tube. The capillary tube cavity was sealed by dipping ends in hot melted wax. Capillary tube set-up was placed in a magnetic field gradient of 294 Gauss/cm for an hour on the microscope stage. Images were taken at 0 h when the magnet was first put into position and at 1 h. We have set an exposure time of 25 ms, magnification at 20x in brightfield.

### Scanning electron microscopy

To prepare SEM samples, 5 μL of the samples were prepared on SEM grid by drop casting method. After incubation at room temperature for 30 min, the samples were dried, and excess samples were removed by blowing nitrogen gas. The images were obtained using Zeiss Sigma 500 SEM (Thornwood, NY, USA).

### Transmission electron microscopy

To prepare the TEM samples, 20 μL of diluted samples in DI water was added to the TED PELLA Formvar/Carbon 200 mesh, copper grid. The sample was allowed to air dry. Images were taken using JEOL 1200 EX II TEM (Tokyo, Japan) operating at 60 kV with a Gatan Orius 600 digital camera.

### HeLa cell culture

The HeLa cells (ATCC CCL-2) were cultured in Dulbecco’s modified Eagle medium (DMEM) supplement with 10% fetal bovine serum (FPS) and 1% penicillin-streptomycin solution at 37 °C in a 5% CO_2_ incubator. The cells were grown on the 35 mm glass bottom microwell dishes (MatTek Corp.) in 2 mL DMEM until DMEM reached about 80% confluence. HeLa cells were cultured in a 35 mm petri dish with a piece of coverglass at the bottom of each chamber in the incubation medium (DMEM) for 24 h for cellular uptake studies. Further, MGNS/DOX was added in the incubation medium at a concentration of 100 μg·mL^−1^ in 5% CO_2_ at 37 °C for different incubation time (30 min, 60 min, 90 min and 120 min) under different conditions (with magnet, heat or both magnet and heat). Media was then removed and the cells were washed twice with PBS (pH 7.4), before the coverglass was visualized under a fluorescence microscope. Here we used Image J software to analyze the fluorescence intensity of fluorescence microscopic images of the cells. We have used cells image without DOX for baseline correction and relative fluorescence intensity of treated cells measured by subtraction of baseline. For every measurement fluorescent intensity of five different cells were used, while average three different images were used to analyze ±s.d. and *P*-value by one-way analysis of variance (ANOVA).

### Human neural progenitor cells derived from induced pluripotent stem cell culture and differentiation

Human differentiated neural progenitor cells (NPCs) derived from induced pluripotent stem cells (iPSCs) were cultured on 20 mg·mL^−1^ poly-L-ornithine (PLO) and 5 mg·mL^−1^ laminin (both from Sigma, St. Louis, MO, USA) coated plates as previously described^27^ NPC medium (DMEM/F12/Glutamax media with N-2 supplement, B-27 supplement, Pen/Strep and basic Fibroblast Growth Factor (bFGF)) was used for culturing NPCs. The incubator was set at 37 °C in 5% CO_2_. The medium was changed every 2–3 days, and cells were split with Accutase (Innovative Cell Technologies) when the medium arrived 100% confluence. bFGF was removed from the medium to induce differentiation. NPCs were seeded on PLO/laminin coated plates in NPC growth medium without bFGF. The medium was changed every 2–3 days. Cells were ready to use after they differentiate for 7 days (the differentiation process was confirmed by immunofluorescence to assess the dendrite and axon growth patterns).

### Cell viability

cell viability after nanoparticle treatment was investigated using a 3-[4,5-dimethylthiazol-2-yl]-2,5-diphenyltetrazolium bromide (MTT, Sigma) assay. For the MTT assay, HeLa cells were seeded into 35 mm petri dish at a density of 1×10^4^ per well in 1 000 μL of the medium and were grown overnight. The differentiated neural cells of density 1×10^5^ per well in 1 000 μL were cultured in differentiation media as described above. Both the cells were then incubated with the test samples for 24 h under different conditions such as without MGNS, with MGNS, and with MGNS/DOX. The cells were exposed to different thermal stimulus and external magnetic field. Afterwards, cells were incubated in medium containing 10% of MTT for 4 h. The precipitated formazan violet crystals were dissolved in DMSO and shaken for 5 min. The absorbance was measured at 570 nm with a multi-detection microplate reader (Synergy HT, BioTek Instruments, Winooski, VT, USA), and the absorbance at 490 nm was measured as a reference. The statistical significance of all the data were validated by one-way ANOVA using Origin Pro 8.0 and data presented as a ±s.d. (*n*=3). The statistical signification in terms of *P*-value was≤0.05 (*).

### Imaging and Mechanical Property analysis by AFM

The fixed Hela cells and NPCs were washed with PBS for three times and were washed with pure water and then dried under nitrogen flow. Samples were air dried, rinsed with deionized water to remove salt crystals, and then air dried again before analysis. The images of Hela cells and NPCs were obtained by using AFM probe with a spring constant of 0.02 N·m^−1^ (TR400PSA from Asylum Research, Santa Barbara, CA, USA) in contact mode using a Dimension Hybrid XYZ scanner from Bruker. The images were obtained in air for all samples. Mechanical property of polyacrylamide hydrogel was measured by using a silicon nitride probe (DNP-10, Bruker, Santa Barbara, CA, USA) with a nominal spring constant of 0.06 N·m^−1^ and tip radius of 20 nm. Indentation force curves were recorded using a contact mode AFM and the elastic modulus values were derived by fitting the force curves using a Sneddon model.

## Results

### Characterization of nanoshuttles

**Figure 1:**
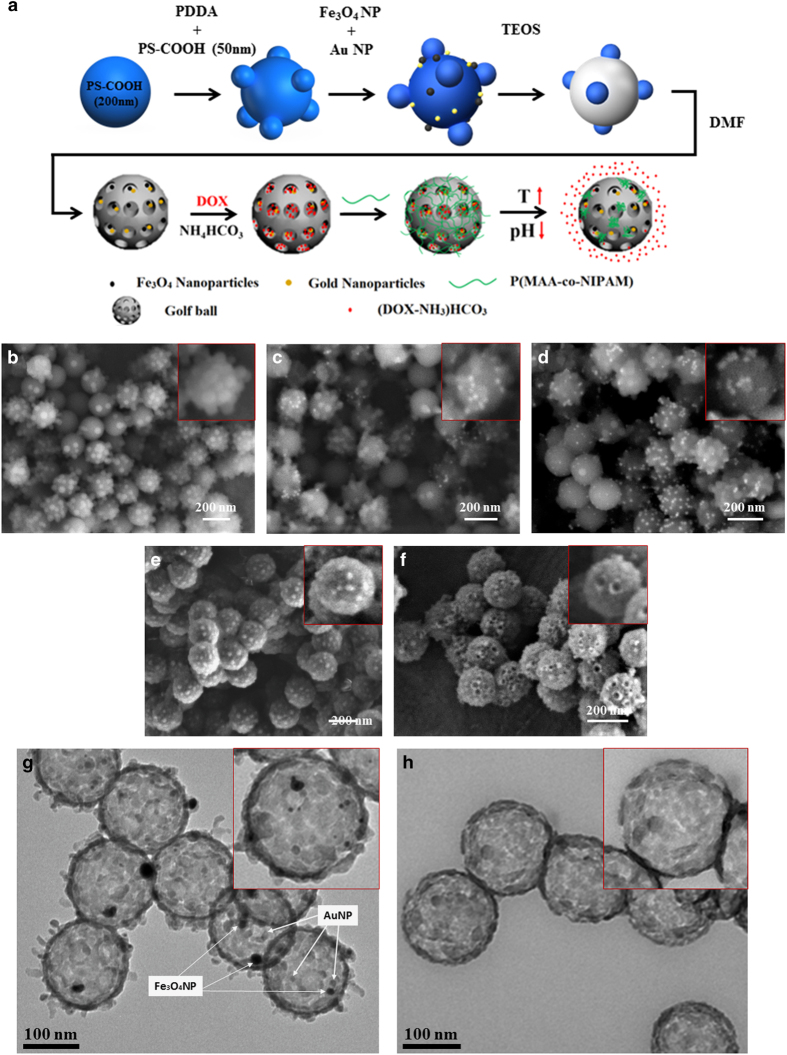
Schematic of P(MAA-co-NIPAM) coated MGNS/DOX. (**a**) The Scheme of DOX loading inside the MGNS and the DOX release; Electron microscopy images of the magnetic and gold particles embedded silica nanoshuttle (MGNS) synthesis process. (**b**) Core-Satellite template, (**c**) Core-Satellite template with iron oxide nanoparticle, (**d**) Core-Satellite template with iron oxide nanoparticle and gold nanoparticle, (**e**) TEOS coating on the template, (**f**) MGNS. (**g**) TEM images of MGNS; (**h**) TEM images of MGNS/DOX.

The Figure 1a showed the schematic diagram of the steps for synthesis of gold and magnetic nanoparticle embedded golf ball, DOX loading, and P(MAA-co-NIPAM) coating. The variation in surface morphology, structure, and size of the magnetic and gold nanoparticles embedded nanoshuttles (MGNSs) during layer-by layer synthesis process was evaluated by SEM and TEM (Figure 1). First, polystyrene templates were synthesized by attaching 0.05 μm polystyrene satellites on PDDA modified polystyrene cores (200 nm) (Figure 1b). Subsequently, iron oxide and gold nanoparticles were electrostatically attached to the template as demonstrated in Figure 1c and d. Next, a silica layer was deposited on the iron oxide and gold embedded template (Figure 1e). The final hollow MGNS morphology was achieved after dissolving polystyrene core and satellites (Figure 1f).

### DOX loading and MGNS encapsulation

**Figure 2:**
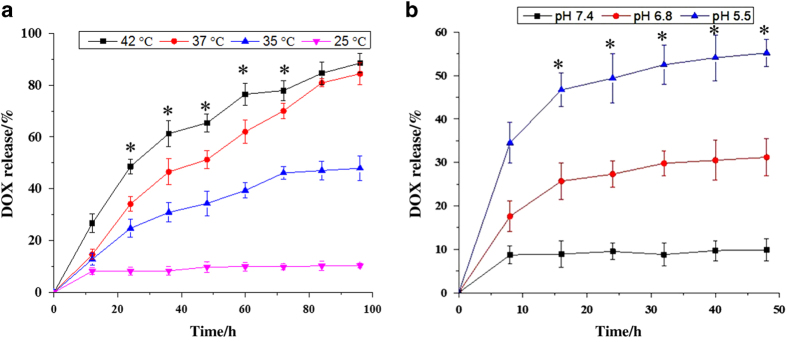
Profiles of Dox release at different temperature for 100 h and at different pH conditions for 50 h (**a**) Dox release from MGNS/DOX system at different temperatures with fixed pH value of 7.4; (**b**) Dox release from MGNS/DOX system at different pH buffer with fixed temperature of 25 °C. The statistical analysis of all the data were performed by one-way analysis of variance (ANOVA) to get ±s.d., *P≤0.05 and *n*=3 for all the analysis.

The efficiency of DOX loading in MGNSs as quantified by DOX fluorescence emission was ~65.8%, and the corresponding drug content was ~36.27 μg·mg^−1^. MGNS pores were then covered using stimuli responsive, polymer P(MAA-co-NIPAM). Upon polymer encapsulation, the size of MGNS increased to 240 nm, and the intensity of the edge of the nanoshuttle became weaker, indicating the presence of the polymer on MGNS. TEM images (Figure 1g and h) of MGNS, further support the SEM analysis (Figures 1c and 2d) of gold and magnetic nanoparticles on the surface of the MGNS.

### Temperature controlled DOX release

The phase transition temperature (*T*_m_) of P(NIPAM-co-MAA) is around 37 °C. Temperature-regulated *in vitro* DOX release from MGNS/DOX was investigated. Four different temperatures, ie, 25°C 35°C, 37°C, and 42 °C were chosen for temperature-dependent drug release experiments. The release curves obtained for four different temperatures at fixed pH value of 7.4 (Figure 2a) show that releasing efficiency of DOX at 42 °C (above *T*_m_) reached ~90% after 96 h, and at 37 °C reached nearly 83%. These two releasing efficiency data points were significantly higher than at lower temperatures (eg, 31% at 35 °C and 15% at 25 °C). Continuous DOX release from MGNS was observed at *T*_m_ or higher temperatures, and DOX amount released increased with the extension of heating time.

### pH controlled DOX release

The pH-controlled release was evaluated at three different pH—7.4, 6.8, and 5.5, representing the pH of blood, tumor extracellular microenvironment, and endosomes, respectively. A microplate reader was used to determine the amount of DOX release from the MGNS at certain time intervals within 48 h at 25 °C. As shown in Figure 2b, the amounts of released DOX increased proportionally with pH. Only about 9% of total DOX was released at pH 7.4. It increased to 31% of total DOX at pH 6.8, and 56% at pH 5.5. Therefore, the amount of DOX released at pH 5.5 was nearly six times higher than that at pH 7.4.

### pH and temperature-controlled DOX release

**Figure 3:**
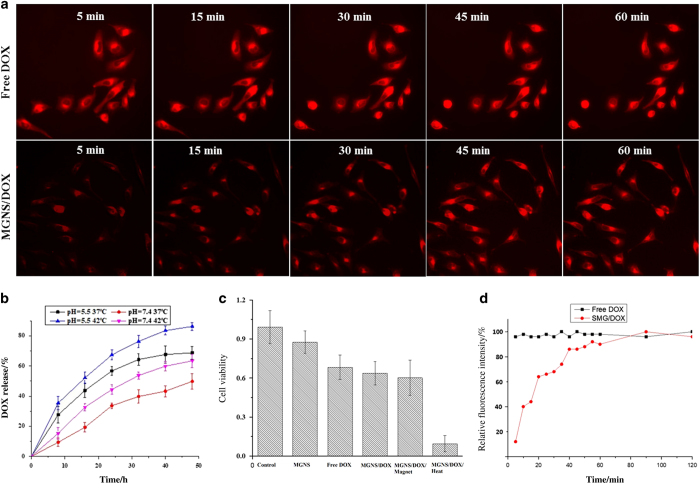
*In vitro* studies of Dox release in Hela cells. (**a**) Fluorescence images of HeLa cells exposed to the free DOX and MGNS/DOX nanoparticles at exposure different time; (**b**) Dox release of MGNS/DOX in PBS buffer at different temperature and different pH; (**c**) Cell viability of HeLa cell co-culture with or without MGNS under different conditions; (**d**) the normalized fluorescence intensity of the free DOX and MGNS/DOX fluorescence images. The statistical analysis of all the data were performed by one-way analysis of variance (ANOVA) to get ±s.d., *P≤0.05 and *n*=3 for all the analysis.

Dual (pH and temperature) stimuli-triggered DOX releasing profiles from the MGNS/DOX drug carrier system were investigated. Four different pH and temperature conditions were chosen to analyze DOX release. These are at pH 7.4, (1) *T*=37 °C, (2) *T*=42 °C, and at pH 5.5, (3) *T*=37 °C, and (4) *T*=42 °C. The first set of conditions simulated the conditions of the normal tissues, while the latter three sets could simulate the conditions of the tumor microenvironment characterized by higher temperatures and acidic conditions. Results described in Figure 3b shows that at 37 °C, DOX release is 68.7% in pH 5.5 and reduced to only about 49.8% at pH 7.4 after 48 h. Although 37 °C is lower than T_m_ at pH 7.4, an acidic pH (pH=5.5) able to stimulate polymer condensation, causing pore opening and drug release. An increase in temperature to the *T*_m_ (37 °C) at pH 7.4, leads to an increased releasing efficacy of 63.4%. An 86.2% DOX release at pH 5.5 and T=42 °C is higher than that at pH 5.5 and *T*=37 °C is potentially due to the combined effect of a working temperature (that is, 42 °C) higher than *T*_m_ and acidic pH.

### HeLa cells’ viability under different conditions

Cytotoxicity of MGNS was determined by the MTT assay. As shown in Figure 3c, DOX loaded MGNS show cytotoxicity similar to that of free DOX. However, external magnetic field-guided delivery of MGNS/DOX/Magnet showed higher cytotoxicity than MGNS/DOX or free DOX. The reason is that the application of external magnetic field promotes MGNS mediated higher amount of DOX delivery into HeLa cells compared to DOX without MGNS. Further, we analyzed the effect of thermal stimuli on drug release and subsequent cytotoxicity. MGNS/DOX under heat treatment shows the highest cytotoxicity among all tested samples, potentially due to the heat-mediated accelerated release of the DOX.

### Intracellular drug release in HeLa cells

We compared HeLa cell drug uptake between free DOX and MGNS/DOX drug delivery methods (Figure 3a and d). We observed a very rapid uptake of free DOX by HeLa cells: after exposure for 5 min, the bright red fluorescence of DOX was readily present in the cell nucleus, and no increase was observed in the next 1 h. However, when HeLa cells were exposed to the MGNS/DOX, only very weak red fluorescence was observed after 5 min that gradually increased over 1 h. After 60 min of exposure, the fluorescence intensity becomes equivalent to the maximum level of the free DOX uptake by HeLa cells.

**Figure 4:**
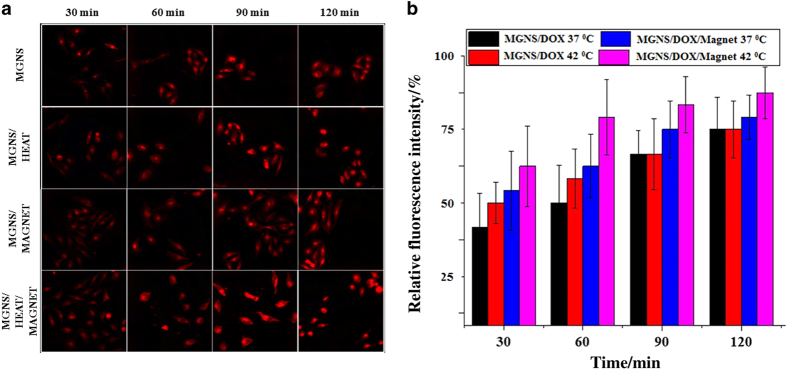
*In vitro* experiments of cellular uptake of MGNS/DOX in HeLa cell. (**a**) Fluorescence images of HeLa cell uptake of MGNS/DOX under different conditions at different time; (**b**) Normalized fluorescence intensity of the fluorescence images of Hela cells’ uptake of MGNS/DOX under different conditions at different time. The statistical analysis of all the data were performed by one-way analysis of variance (ANOVA) to get ±s.d., *P≤0.05 and *n*=3 for all the analysis.

Fluorescence of DOX-loaded MGNS co-cultured with human HeLa cells were recorded for different time points. The results are shown in Figure 4a. After 30 min of drug exposure, we observed a relatively weak fluorescence within the HeLa cells, and there was no obvious difference among four different conditions. The fluorescence intensity of HeLa cells did not increase significantly with time if there was no thermal or pH stimuli. For the other three samples (with heat, with a magnet, with heat and magnet), the fluorescence intensity increased significantly at 60 min compared with the 30 min culture. After 90 min of drug exposure, the cells without treatment appear to keep nearly the same fluorescence intensity while other three samples showed amplified fluorescence intensity due to the increase of the intracellular DOX concentration (Figure 4b). The increase of fluorescence intensity seems the most apparent for the samples with the combined application of the heat and magnetic field after 120 min of exposure. However, the control cell cultures (ie co-culture with MGNS and without exposure to magnetic field or heat or DOX), showed similar fluorescence intensity at different time point (data not shown). It was observed that the MGNS were mainly accumulated in the nucleus of HeLa cells. Figure 4b shows relative average fluorescence intensities of single cells for a clearer comparison.

### Intracellular drug release in NPCs

**Figure 5:**
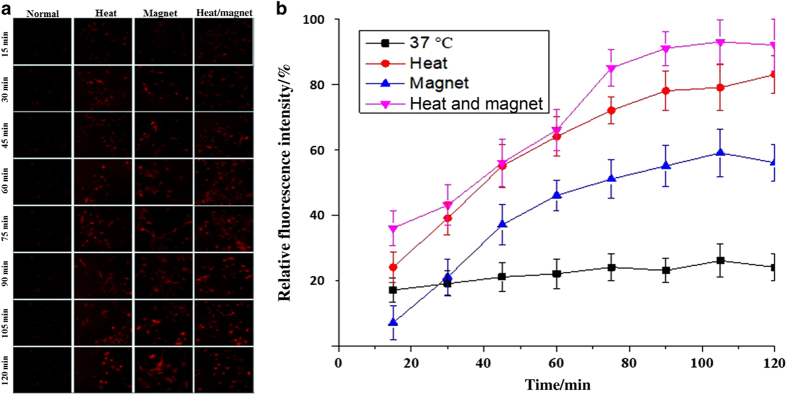
*In vitro* cellular uptake of MGNS/Dox in human neuronal progenitor cells (NPCs). (**a**) Fluorescence images of human NPC uptake of MGNS/DOX under different condition at different time; (**b**) Normalized fluorescence intensity of fluorescence images of the NPCs’ MGNS/DOX uptake. The statistical analysis of all the data were performed by one-way analysis of variance (ANOVA) to get ±s.d., *P≤0.05 and *n*=3 for all the analysis.

We wanted to investigate whether there is a difference of the DOX release between epithelial cells and non-transformed human cells of distinct histogenesis, neural progenitor cells (NPCs), and to observe how the live human neurons will respond to the DOX releases. For cells without stimuli, the changes in fluorescence intensity showed similar tendency to the HeLa cells: there was no obvious increase in the intensity at 30, 60, 90 and 120 min (Figure 5). However, for the other three conditions, the MGNS-exposed NPCs showed the increase in the fluorescence intensity with the increased exposure time (Figure 5b). Similar to the HeLa cells, the NPCs exposed to combined heat and magnetic field showed the highest fluorescence intensity. MGNS-exposed NPCs with the magnetic field and heat treatments showed much higher increase in fluorescence intensity compared to HeLa cells after 90 min. In NPC experiments, a higher level of fluorescence intensity in the treated cells compared with the corresponding untreated (control) cells (Figure 5a) was observed. After 15 min of NPCs exposure to MGNS/DOX, the heat-treated sample and both heat- and magnetic field-treated samples already showed increased fluorescence intensity, while the only magnetic field-treated sample and untreated control samples showed no changes. We observed the rapid enhancement of fluorescence intensity for NPCs exposed to MGNS for 30 min to 90 min interval, while continuing incubation from 90 min to 120 min showed relatively slow increase in the fluorescence intensity of all the samples. At all examined experimental time points, comparisons of the cells cultured under three treatment regimens revealed that the heat-treated and dual stimuli-treated cells (cells’ subjected to both heat- and magnetic field-treatments) showed much higher fluorescence intensity than the cells treated with the magnetic field only (Figure 5b).

### AFM images of MGNS/DOX uptake in HeLa cells

**Figure 6:**
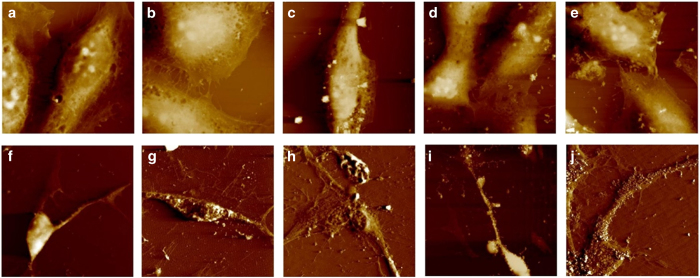
AFM images of MGNS/DOX uptake in HeLa cells and human neuronal progenitor cells (NPCs). AFM images of the uptake in Hela cells at (**a**) 0 min, (**b**) 30 min, (**c**) 60 min, (**d**) 90 min, (**e**) 120 min. AFM images of MGNS/DOX uptake in NPCs at (**f**) room temperature, (**g**) 37 °C, (**h**) 39 °C, (**i**) room temperature with magnet and (**j**) 39 °C with magnet.

*In vitro* effect of MGNS/DOX on cellular morphology as a function of time was analyzed by AFM. From the AFM images of HeLa cells without MGNS/DOX treatment we observed that HeLa cells presented a distinct and clear shape, and we could see the clearly visible nucleus in the cells (Figure 6a). After exposure to the MGNS/DOX for 30 min (Figure 6b), the HeLa cells maintained extended morphology and pseudopods at the edge of the cell membrane were distinctly visible. Nanoclusters in the cells’ cytoplasm and around the nucleus were observed. The whole cell outline and some cell’s skeleton under the cell membrane were visible. After exposure for 60 min (Figure 6c), the cell membrane outline was still visible, but the pseudopods and cell’s cytoplasm appeared to shrink. Some peripheral ruffles and small globular structures were resolved at nanometer-scale resolution. At this time point, more nanoparticles appeared to be in the cytoplasm and DOX localized in the cell’s nucleus. After 90 min (Figure 6d), the HeLa cells’ morphology changed, as the pseudopods around the cell membrane were less apparent, and even more, nanoparticles were observed inside the HeLa cell. After 120 min (Figure 6e), the morphology of HeLa cells changed markedly as they displayed an irregular shape. The edge between the cells’ nucleus and cytoplasm was visible, and many more nanoparticles were embedded inside the cells. The cells’ surface was complex with a large number of ruffles. Because of the disruption of the ruffles, the cell skeletons could not be resolved. The whole cells were not highly extended anymore.

### AFM images of MGNS/DOX uptake in differentiated neural cells

As seen in Figure 6f–j, NPCs at room temperature had a regular shape, and their surface was relatively smooth (Figure 6f). Cytoskeleton structures surrounded the nucleus and some filopodia extended from the tip of the growth cone. Following treatment with MGNS/DOX MGNS at 37 °C (Figure 6g), the neural cell still kept regular shape, and the cell surface still appeared intact. Some nanoshuttles were observed inside the cell. For the sample exposed to the heat, the surface morphology changed significantly after 2 h of heat treatment (Figure 6h). The neural cells appeared to have shrunk, the edges of neural cells became irregular, and the more nanoparticles were embedded in the cells. For the neural cells with magnetic field exposure for 2 h, neural cells shrank more markedly. We could see an increased number of MGNS gathered inside the neural cell and some filopodia disappeared and shrank (Figure 6i). In the samples with combined exposure to the heat and magnet, many more MGNS were inside the neural cells, and clearly defined cytoskeleton structures surrounding the nucleus, and obvious pores appeared on the surface of the neural cells’ membrane (Figure 6j). The membrane surface features became more unevenly distributed.

### MGNS transport in porous materials

**Figure 7:**
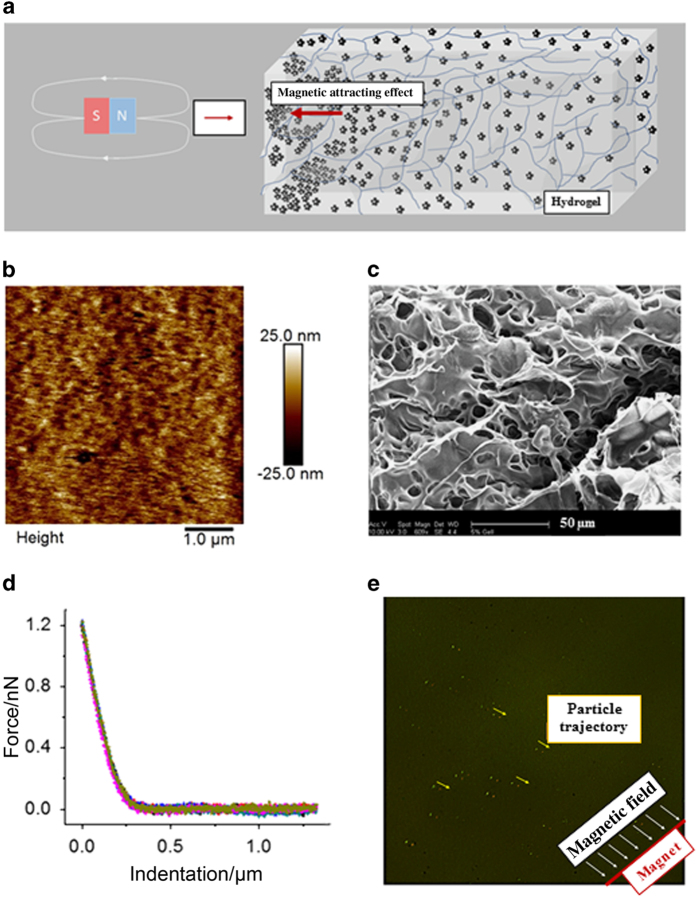
Schematic representation of the polyacrylamide gel (PAG) experiment with MGNS and characteristics of PAG. (**a**) Schematic of the MGNS transport in PAG, (**b**) AFM image of PAG, (**c**) SEM image of PAG, (**d**) Mechanical property of polyacrylamide hydrogel by AFM analysis using silicon nitride probe (DNP-10, Bruker), (**e**) External magnetic field mediated transport of MGNS in PAG.

**Figure 8:**
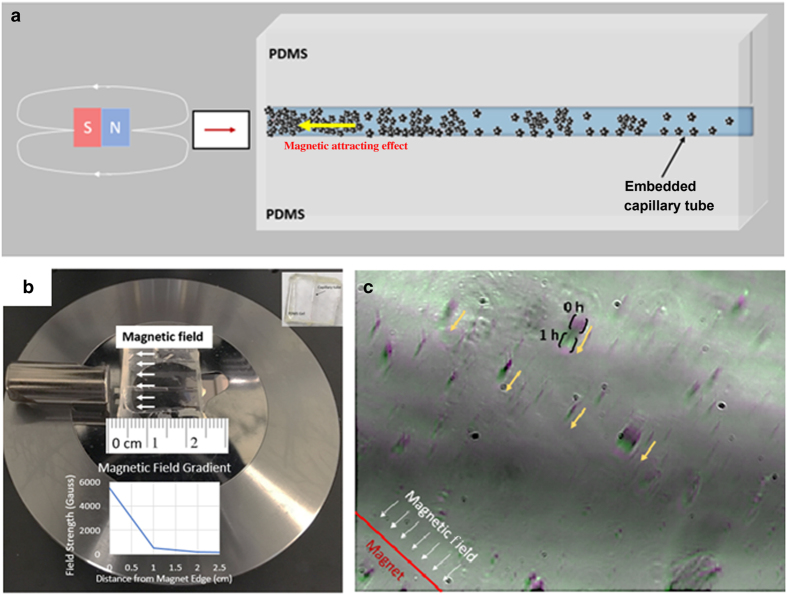
External magnetic field-guided transport of MGNS in a capillary tube. (**a**) Schematic for magnetic pulling; (**b**) Set-up of capillary tube and magnet of microscope stage; (**c**) Brightfield of nanocarrier cluster movement over an hour.

MGNS transport was monitored in porous material and capillary tube under external magnetic field (Figures 7 and 8). The transport and delivery of MGNS in porous material was analyzed to test the relevance of MGNS in composite tissue-like porous environment, including bone (Figure 7a). For this analysis, 5% polyacrylamide gel (PAG) was used. Mechanical properties of polyacrylamide hydrogel was analyzed by AFM using silicon nitride probe (DNP-10, Bruker) with a nominal spring constant of 0.06 N·m^−1^ and tip radius of 20 nm. The force curve was measured in deionized water. The elastic modulus was measured and fitted using the Sneddon modulus. The results showed that hydrated PAG has elastic modulus of ~60 kPa (*n*=7) while dry sample has very high elastic modulus (~700 kPa) (Figure 7d). SEM and AFM analyses showed that PAG has large pore with an average size of ~18 μm (Figure 7b and c). The directional transport of MGNS in the presence of external magnetic field of 500 Gauss per cm in PAG was observed; ~3 μm diameter clusters of MGNS showed displacement of ~15 μm with the speed of ~4 μm·h^−1^ (Figure 7e).

Similarly, the displacement of MGNS clusters in capillary tube was monitored over an hour. The purpose of these experiments was to simulate MGNS transport in blood capillary. The results clearly indicated that the external field mediated directional displacement of MGNS with time (Figure 8a–c). The particle cluster of ~1400 μm^2^ showed displacement of ~14 μm in an hour in an external magnetic field of 275 Gauss per cm.

## Discussion

MGNS are hybrid smart nanoshuttles that have the capability to encapsulate and release on-demand theranostic agents due to their hollow core and the nanopores in the shell. The MGNS design makes biomolecules smaller than the size of the pore suitable cargo for targeted theranostics’ applications. We demonstrate that external stimuli-responsive polymer P(MAA-co-NIPAM)-coating on MGNS acts as an efficient gatekeeper to prevent the spontaneous DOX release. This property of P(NIPAM-co-MAA) is the result of its temperature and pH sensitivity.^29–32^ At a temperature below the phase transition temperature (*T*_m_=37 °C), the polymer is in hydrogel state and stretched, and the pores on the MGNS are covered completely. In contrast, when the temperature raised above 37 °C, the polymer becomes condensed and opens the pores. In acidic conditions, the DOX precipitate (DOX-NH_3_)HCO_3_ decomposes, thereby allowing controlled DOX release. As seen in Figure 2a, at temperatures below *T*_m_ low fluorescent readings are observed. This is because pores on the MGNS are fully covered by the P(NIPAM-co-MAA) polymer, and DOX remains in its precipitated state of (DOX-NH_3_)HCO_3_ due to less solubility in aqueous solution. Increase of temperature is associated with increased fluorescent readings reflecting DOX release. Therefore, P(NIPAM-co-MAA) coated MGNS is shown to manifest thermo-responsive drug release.

Similarly, pores are also opened when the pH is decreased to acidic conditions.^33,34^ Higher DOX release at acidic pH is due to pH sensitivity of the MAA moiety in P(NIPAM-co-MAA) polymer; the polymer changes from hydrophilic to hydrophobic state with decrease in pH. There is little or no DOX released in neutral pH conditions because the polymer exhibits an extended hydrophilic state that covers MGNS pores. In contrast, at acidic pH, the polymer changes to a hydrophobic state and was contracted, thus opening the pores resulting in higher fluorescent signals in Figure 2b. Dual stimuli-triggered drug release profiles demonstrate dynamics potentially relevant to complex physiological conditions *in vivo*. At the sites of inflammations and in solid tumor tissues across all organs and structures including brain, heart, lung and bone, typically characterized by acidic pH, the polymer-coated nanoshuttles would respond by polymer condensation following the temperature increase, causing pore opening and drug release. Additionally, MTT assay results show that MGNS have good cyto-compatibility, indicating that subsequent decrease in cell viability (Figure 3c) are due to the presence of the released drug, DOX, in response to combined effect of external stimuli (heat, pH, and external magnetic field).

We have reported previously that MGNS/DOX drug delivery system showed lysosome-mediated fast release of a drug due to lower pH (that is, 5) in lysosomes.^35^ Here we showed that MGNS/DOX could potentially deliver and maintain a high concentration of DOX in HeLa and in differentiated neural cells. From analysis of fluorescence intensity profiles HeLa cells (Figure 3a and d) and NPC cells (Figure 5a and b), the effect of thermal stimuli and magnetic field on the intracellular DOX release is evident in both the cells. It was evident that dual stimuli (thermal/pH and magnetic field) further enhanced the release (Figure 4). This suggests that combining the responsiveness to two stimuli should increase the therapeutic effect due to enhanced drug release. More importantly, results demonstrated that MGNS nanoshuttles could potentially deliver and release much more drugs selectively at tumor sites. Application of the magnetic field localized MGNS in the cells and enhanced the MGNS/DOX entry inside cells while increased temperature increased local temperature due to presence of gold and iron oxide and enhanced the release. This conclusion is supported with AFM data that show that MGNS readily enter HeLa cells and the number of particles inside the cell increased with time. Consequently, the cell morphology changed to irregular shape after 120 min of exposure to the DOX-loaded nanoparticles. Intracellular uptake experiments with NPC indicated that the NPCs exposed to the DOX-loaded MGNS appear more sensitive to the external stimuli than HeLa cells. This is consistent with AFM images that show higher uptake of MGNS in NPC (Figure 6j) than in HeLa cells (Figure 6e). All the AFM images demonstrate that the MGNS could readily enter the neural cells and the magnet was instrumental in localizing nanoshuttles in the cell.

Targeted delivery of drug molecules in bone tissue, which is a suitable microenvironment for cancerous cell growth and destination for metastatic cells, is a major challenge. Since MGNS nanoshuttles have the ability to be magnetically guided to a target region, their transport behavior becomes important to study in different environment such as flowing liquid and porous composite materials.. As the bone is a composite material, the stiffness varies dramatically among various sub-structures. In addition, it also varies with the age and disease condition such as normal human Menisci (176–205 kPa), aged human menisci (274–300 kPa), and aged osteoarthritis (364–401 kPa).^36^ Nanoparticles made up of PLGA, PEG and bisphosphonate have been used to control delivery of therapeutic agent to bone. Nanoparticle based drug carrier provide efficient delivery method for treatment of cancer growth in bone tissue.^37^ Nanoparticles used with scaffolds are advantageous because they not only provide targeted drug delivery potential but also protect bioactive agents, and reduced the side effects.^38^ Fast evolution of multifunctional nanostructures will help controlling drug delivery and treatment of bone specific disease and regeneration.^39^ Moreover, functionalized nanoparticles can be used for delivery of active biomolecules and can help to repair bone fracture.^40,41^ In our study, we have used 5% polyacrylamide gel (PAG) as a model to analyze the transport of MGNS. It was observed that wet and dry PAG samples have a range of elastic modulus in the range of soft-to hard bone tissue. MGNS travel slowly and in clusters in dense gel such as PAG, indicating that they are capable of being directed to a target site, for example, in porous bone tissue. Polymer-coated MGNS travel faster in fluid environments, although in larger clusters. This property could be exploited for intravenous delivery of payload through blood to target areas such as the bone marrow.

Preclinical development of theranostics using our Nanoshuttles, that have potentially broad practical applications, would accelerate implementation in clinical practice of the evidence-based next generation nanomedicine for all tissues and organs, including bone. Specifically, there are several significant promises, including targeted magnetic guidance based on-demand retention at defined anatomic locations of bones. This feature is particularly important for specific hard-to-reach anatomic locations in human body such as internal linings of the skull, other parts of the skeleton, surfaces of bone cavities, and bone marrow. Targeted magnetic guidance will enable passage from blood through biological barriers such as blood–brain barrier and/or blood-solid tissues barriers. Moreover, intrinsically embedded features enable the real-time imaging of tissues and this feature is particularly important to extend the potential utility of this technology from delivery to theranostic application.

## Conclusions

Magnetic and gold nanoparticles embedded nanoshuttle was designed and used as a drug carrier in human cells to demonstrate its uptake and controlled release of the payload. We loaded DOX inside the MGNS using the precipitation method and utilized thermal/pH bi-sensitive polymer P(NIPAM-co-MAA) as a gatekeeper to nanopores. Fluorescence signal shows significantly high loading efficiency of DOX as well as pH and temperature controlled release. *In vitro,* drug release experiments in HeLa cells and neurons indicated that we could effectively control the DOX release inside the cells by applying heat and magnetic field. MGNS/DOX can be concentrated and retained inside human cells, and DOX can be released in a controlled manner with high efficiency. Human neurons (ie, NPCs) showed more responsiveness to the control external stimuli compared with the HeLa cells. These differential drug release responses of MGNS in HeLa cells and NPCs show a promise for the design of cell specific controlled drug release systems. Moreover, a use of the magnetic field to pull MGNS holds a great potential for tissue specific (eg, bone, heart, lung, and brain) clinical applications in targeted drug delivery and on-demand drug release.

## References

[bib1] Wang C, Zhang K, Zhou Z et al. Vancomycin-modified Fe_3_O_4_@ SiO_2_@ Ag microflowers as effective antimicrobial agents. Int J Nanomed 2017; 12: 3077. 10.2147/IJN.S132570 PMC5399987 28450783

[bib2] Zhao S, Li J, Cao D et al. Recent advancements in flexible and stretchable electrodes for electromechanical sensors: strategies, materials, and features. ACS Appl Mater Interfaces 2017; 9: 12147–12164. 28281337 10.1021/acsami.6b13800

[bib3] Pitakchatwong C, Chirachanchai S. Thermo-magnetoresponsive dual function nanoparticles: an approach for magnetic entrapable-releasable chitosan. ACS Appl Mater Interfaces 2017; 9: 10398–10407. 28256121 10.1021/acsami.6b14676

[bib4] Sun Q, You Q, Pang X et al. A photoresponsive and rod-shape nanocarrier: Single wavelength of light triggered photothermal and photodynamic therapy based on AuNRs-capped & Ce6-doped mesoporous silica nanorods. Biomaterials 2017; 122: 188–200. 28131043 10.1016/j.biomaterials.2017.01.021

[bib5] Wu C, Gong MQ, Liu BY et al. Co-delivery of multiple drug resistance inhibitors by polymer/inorganic hybrid nanoparticles to effectively reverse cancer drug resistance. Colloids Surf B 2017; 149: 250–259. 10.1016/j.colsurfb.2016.10.029 27768915

[bib6] Yi H, Ur Rehman F, Zhao C et al. Recent advances in nano scaffolds for bone repair. Bone Res 2016; 4: 16050. 28018707 10.1038/boneres.2016.50 PMC5153570

[bib7] Vieira S, Vial S, Reis RL et al. Nanoparticles for bone tissue engineering. Biotechnol Prog 2017; 33: 590–611. 28371447 10.1002/btpr.2469

[bib8] Gu W, Wu C, Chen J et al. Nanotechnology in the targeted drug delivery for bone diseases and bone regeneration. Int J Nanomed 2013; 8: 2305–2317. 10.2147/IJN.S44393 PMC3699134 23836972

[bib9] Lockhart JN, Beezer DB, Stevens DM et al. One-pot polyglycidol nanogels via liposome master templates for dual drug delivery. J Control Release 2016; 244: 366–374. 27411978 10.1016/j.jconrel.2016.07.013

[bib10] Viger ML, Collet G, Lux J et al. Distinct ON/OFF fluorescence signals from dual-responsive activatable nanoprobes allows detection of inflammation with improved contrast. Biomaterials 2017; 133: 119–131. 28433935 10.1016/j.biomaterials.2017.03.042 PMC5704950

[bib11] Babu A, Munshi A, Ramesh R. Combinatorial therapeutic approaches with RNAi and anticancer drugs using nanodrug delivery systems. Drug Dev Ind Pharm 2017; 43: 1391–1401. 28523942 10.1080/03639045.2017.1313861 PMC6101010

[bib12] Liao SH, Liu CH, Bastakoti BP et al. Functionalized magnetic iron oxide/alginate core-shell nanoparticles for targeting hyperthermia. Int J Nanomed 2015; 10: 3315. 10.2147/IJN.S68719 PMC4427608 26005343

[bib13] Wang H, Zhu W, Huang Y et al. Facile encapsulation of hydroxycamptothecin nanocrystals into zein-based nanocomplexes for active targeting in drug delivery and cell imaging. Acta Biomater 2017; 61: 88–100. 28433787 10.1016/j.actbio.2017.04.017

[bib14] Kawasaki R, Sasaki Y, Katagiri K et al. Magnetically guided protein transduction by hybrid nanogel chaperones with iron oxide nanoparticles. Angew Chem, Int Ed 2016; 55: 11377–11381. 10.1002/anie.201602577 27295070

[bib15] Lee N, Yoo D, Ling D et al. Iron oxide based nanoparticles for multimodal imaging and magnetoresponsive therapy. Chem Rev 2015; 115: 10637–10689. 26250431 10.1021/acs.chemrev.5b00112

[bib16] Casciaro B, Moros M, Rivera-Fernández S et al. Gold-nanoparticles coated with the antimicrobial peptide esculentin-1a(1-21)NH_2_ as a reliable strategy for antipseudomonal drugs. Acta Biomater 2017; 47: 170–181. 27693686 10.1016/j.actbio.2016.09.041

[bib17] Feng Q, Shen Y, Fu Y et al. Self-assembly of gold nanoparticles shows microenvironment-mediated dynamic switching and enhanced brain tumor targeting. Theranostics 2017; 7: 1875–1889. 28638474 10.7150/thno.18985 PMC5479275

[bib18] Gao H, Goriacheva OA, Tarakina NV et al. Intracellularly biodegradable polyelectrolyte/silica composite microcapsules as carriers for small molecules. ACS Appl Mater Interfaces 2016; 8: 9651–9661. 27008032 10.1021/acsami.6b01921

[bib19] Ahmad MZ, Akhter S, Rahman Z et al. Chapter 16 – nanomedicine based drug targeting in alzheimer’s disease: high impact of small carter. Drug Design & Discovery in Alzheimers Disease 2014; 716–739.

[bib20] Le PN, Nguyen NH, Nguyen CK et al. Smart dendrimer-based nanogel for enhancing 5-fluorouracil loading efficiency against MCF7 cancer cell growth. Bull Mater Sci 2016; 39: 1493–1500.

[bib21] Zazo H, Colino CI, Warzecha KT et al. Gold nanocarriers for macrophage-targeted therapy of human immunodeficiency virus. Macromol Biosci 2017; 17. 10.1002/mabi.201600359 27748547

[bib22] Wen CY, Xie HY, Zhang ZL et al. Fluorescent/magnetic micro/nano-spheres based on quantum dots and/or magnetic nanoparticles: preparation, properties, and their applications in cancer studies. Nanoscale 2016; 8: 12406–12429. 26831217 10.1039/c5nr08534a

[bib23] Nan X, Zhang X, Liu Y et al. Dual-targeted multifunctional nanoparticles for magnetic resonance imaging guided cancer diagnosis and therapy. ACS Appl Mater Interfaces 2017; 9: 9986–9995. 28263051 10.1021/acsami.6b16486

[bib24] Xu Y, He R, Lin D et al. Laser beam controlled drug release from Ce6-gold nanorod composites in living cells: a FLIM study. Nanoscale 2015; 7: 2433–2441. 25565649 10.1039/c4nr05574h

[bib25] Tehrani MD, Yoon JH, Kim MO et al. A novel scheme for nanoparticle steering in blood vessels using a functionalized magnetic field. IEEE Trans Biomed Eng 2015; 62: 303–313. 25163053 10.1109/TBME.2014.2351234

[bib26] Zhao C, Liu X, Zhang X et al. A facile one-step method for preparation of Fe_3_O_4_/CS/INH nanoparticles as a targeted drug delivery for tuberculosis. J Mater Sci Eng C 2017; 77: 1182–1188. 10.1016/j.msec.2017.03.137 28531994

[bib27] Kassan A, Egawa J, Zhang Z et al. Caveolin-1 regulation of disrupted-in-schizophrenia-1 as a potential therapeutic target for schizophrenia. J Neurophysiol 2017; 117: 436–444. 27832597 10.1152/jn.00481.2016 PMC5253400

[bib28] Hang L, Li C, Zhang T et al. A novel process to prepare a thin silica shell on the PDDA-stabilized spherical Au nanoparticles assisted by UV light irradiation. RSC Adv 2014; 4: 64668–64674.

[bib29] Gao X, Cao Y, Song X et al. pH- and thermo-responsive poly(N-isopropylacrylamide-co-acrylic acid derivative) copolymers and hydrogels with LCST dependent on pH and alkyl side groups. J Mater Chem B 2013; 1: 5578–5587. 10.1039/c3tb20901f 32261182

[bib30] Ma X, Liu Q. Preparation of poly(N-isopropylacrylamide)-block-(acrylic acid)-encapsulated proteinaceous microbubbles for delivery of doxorubicin. Colloids Surf B 2017; 154: 115–122. 10.1016/j.colsurfb.2017.03.019 28334688

[bib31] Son KH, Lee JW. Synthesis and characterization of poly(Ethylene Glycol) based thermo-responsive hydrogels for cell sheet engineering. Materials 2016; 9: 854. 10.3390/ma9100854 PMC5456593 28773974

[bib32] Zhang J, Peppas NA. Synthesis and characterization of pH- and temperature-sensitive poly(methacrylic acid)/poly(N-isopropylacrylamide) interpenetrating polymeric networks. Macromolecules 2000; 33: 102–107.

[bib33] Karg M, Pastoriza-Santos I, Rodriguez-González B et al. Temperature, pH, and ionic strength induced changes of the swelling behavior of PNIPAM-poly(allylacetic acid) copolymer microgels. Langmuir 2008; 24: 6300–6306. 18489184 10.1021/la702996p

[bib34] Al-Manasir N, Zhu K, Kjøniksen AL et al. Effects of temperature and pH on the contraction and aggregation of microgels in aqueous suspensions. J Phys Chem B 2009; 113: 11115–11123. 19618921 10.1021/jp901121g

[bib35] Ramachandran S, Quist AP, Kumar S et al. Cisplatin nanoliposomes for cancer therapy: AFM and fluorescence imaging of cisplatin encapsulation, stability, cellular uptake, and toxicity. Langmuir 2006; 22: 8156–8162. 16952256 10.1021/la0607499

[bib36] Kwok J, Grogan S, Meckes B et al. Atomic force microscopy reveals age-dependent changes in nanomechanical properties of the extracellular matrix of native human menisci: implications for joint degeneration and osteoarthritis. Nanomedicine 2014; 10: 1777–1785. 24972006 10.1016/j.nano.2014.06.010 PMC4374607

[bib37] Swami A, Reagan MR, Basto P et al. Engineered nanomedicine for myeloma and bone microenvironment targeting. Proc Natl Acad Sci USA 2014; 111: 10287–10292. 24982170 10.1073/pnas.1401337111 PMC4104924

[bib38] Monteiro N, Martins A, Reis RL et al. Nanoparticle-based bioactive agent release systems for bone and cartilage tissue engineering. Regener Ther 2015; 1: 109–118. 10.1016/j.reth.2015.05.004 PMC6581799 31245450

[bib39] Yang L, Webster TJ. Nanotechnology controlled drug delivery for treating bone diseases. Expert Opin Drug Delivery 2009; 6: 851–864. 10.1517/17425240903044935 19637973

[bib40] Wang Q, Yan J, Yang J et al. Nanomaterials promise better bone repair. Mater Today 2016; 19: 451–463.

[bib41] Liang C, Guo B, Wu H et al. Aptamer-functionalized lipid nanoparticles targeting osteoblasts as a novel RNA interference-based bone anabolic strategy. Nat Med 2015; 21: 288–294. 25665179 10.1038/nm.3791 PMC5508976

